# A method for small-sized wheat seedlings detection: from annotation mode to model construction

**DOI:** 10.1186/s13007-024-01147-w

**Published:** 2024-01-29

**Authors:** Suwan Wang, Jianqing Zhao, Yucheng Cai, Yan Li, Xuerui Qi, Xiaolei Qiu, Xia Yao, Yongchao Tian, Yan Zhu, Weixing Cao, Xiaohu Zhang

**Affiliations:** 1 National Engineering and Technology Center for Information Agriculture, Nanjing Agricultural University, Nanjing, 210095 China; 2 College of Geography, Jiangsu Second Normal University, Nanjing, 211200 China; 3 Key Laboratory for Crop System Analysis and Decision Making, Ministry of Agriculture and Rural Affairs, Nanjing, 210095 China; 4 Jiangsu Key Laboratory for Information Agriculture, Nanjing, 210095 China; 5 Jiangsu Collaborative Innovation Center for Modern Crop Production, Nanjing, 210095 China

**Keywords:** Wheat seedlings detection, Local annotation, Unmanned aerial vehicle (UAV) images, YOLO

## Abstract

The number of seedlings is an important indicator that reflects the size of the wheat population during the seedling stage. Researchers increasingly use deep learning to detect and count wheat seedlings from unmanned aerial vehicle (UAV) images. However, due to the small size and diverse postures of wheat seedlings, it can be challenging to estimate their numbers accurately during the seedling stage. In most related works in wheat seedling detection, they label the whole plant, often resulting in a higher proportion of soil background within the annotated bounding boxes. This imbalance between wheat seedlings and soil background in the annotated bounding boxes decreases the detection performance. This study proposes a wheat seedling detection method based on a local annotation instead of a global annotation. Moreover, the detection model is also improved by replacing convolutional and pooling layers with the Space-to-depth Conv module and adding a micro-scale detection layer in the YOLOv5 head network to better extract small-scale features in these small annotation boxes. The optimization of the detection model can reduce the number of error detections caused by leaf occlusion between wheat seedlings and the small size of wheat seedlings. The results show that the proposed method achieves a detection accuracy of 90.1%, outperforming other state-of-the-art detection methods. The proposed method provides a reference for future wheat seedling detection and yield prediction.

## Introduction

Wheat is one of the major staple crops worldwide and plays an essential role in food security. The number of seedlings is a crucial indicator of the plant population during the seedling stage, affecting grain structure and wheat yield to some extent. Therefore, counting wheat seedlings has become important in wheat production management [1, 2]. Traditional seedling counting methods rely on manual field surveys with low counting efficiency [3]. With the rapid development of artificial intelligence technology, object detection methods based on deep learning have been applied to wheat seedling counting [4]. Deep learning automatically extracts low-level and high-level features from a large number of image samples, showing better robustness and generalization capabilities. Existing studies have used CNN models to perform wheat seedling detection tasks [5, 6], including two-stage detection methods represented by the Faster-RCNN algorithm and one-stage detection methods represented by the YOLO algorithm [7]. In such studies, researchers often focus on the wheat detection model to improve performance by enhancing the model architecture and loss functions [8, 9]. However, high-quality annotated data has always been crucial in constructing and applying object detection models [10–12]. In previous studies, annotation patterns have been optimized by setting the annotated regions’ size and adjusting the bounding boxes’ orientation to improve the acquisition of annotated data [13–15]. However, individual wheat seedlings are tiny and show significant image morphological variations. Direct annotation of the whole wheat plants results in less information reflecting the characteristics of the seedlings within the annotated bounding boxes. In addition, the interference of the soil background is significant, resulting in low detection efficiency of the model. Some researchers have proposed alternative annotations of key parts, such as leaf tips and local, instead of annotating the whole plant [15]. However, due to the mechanical or drill sowing for wheat, the seedlings have small local sizes and dense distributions during the seedling stage [16]. The small size and dense distribution of wheat seedlings increase the complexity of manual annotation, which is compounded by the presence of significant non-wheat seedling portions of the soil background within the annotation boxes, thereby affecting the robustness of the model [17]. At the same time, current wheat seedling detection methods face challenges in accurately locating and classifying small-sized seedlings. They often suffer from confusion between wheat seedlings and the soil background, making them unsuitable for scenarios characterized by densely distributed wheat seedlings [18, 19]. The combination of poor data annotation and deficiencies in the wheat seedling detection model has resulted in existing methods being unable to meet the requirements for real-time and accurate wheat seedling detection [16, 20].

This study proposes a small wheat seedling detection method based on local annotation and YOLOv5 in unmanned aerial vehicle (UAV) images to solve the above problems. Instead of annotating the whole wheat seedling, which is called global annotation, local annotation of the wheat seedling is used to optimize the annotation mode of the wheat seedling dataset. At the same time, the YOLOv5 is enhanced to improve its detection capability for small objects, thereby realizing wheat seedling detection based on local annotation.

## Materials and methods

**Fig. 1:**
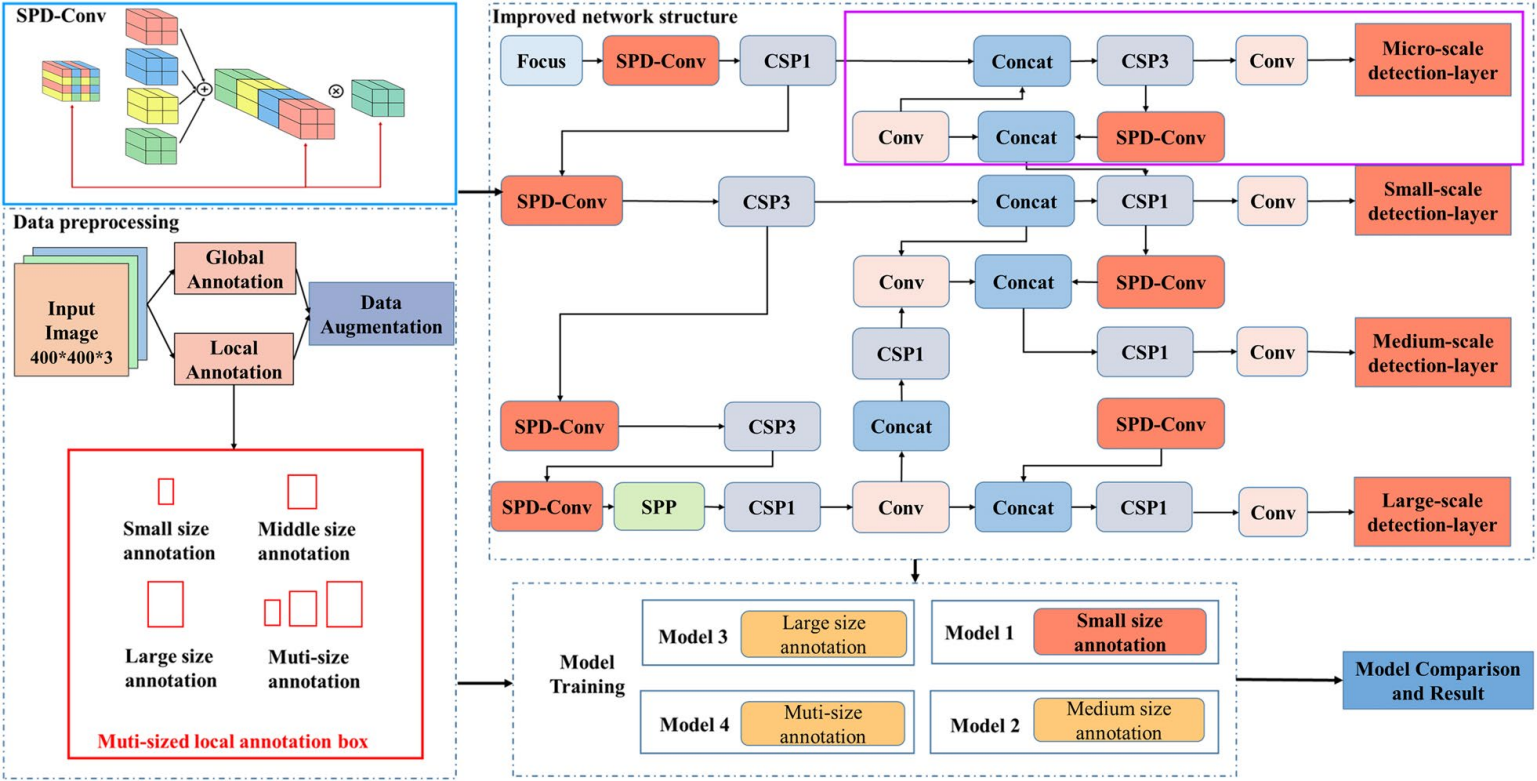
Technical framework. The red solid box represents different annotation modes. The purple solid box represents the newly added micro-scale detection layer. The blue solid box represents the SPD-Conv

This study proposes an optimization method for wheat seedling detection by fusing local annotation mode and improved model structure. Firstly, the collected wheat seedling UAV images were segmented into standard-sized patches. Then, three different sizes of annotation boxes were used to annotate the local regions of the wheat seedling in the images, and the dataset for wheat seedling detection was created. The standard YOLOv5 was used as the baseline model, and it is enhanced by adding a micro-scale layer and incorporating the SPD-Conv module (Fig. 1). These enhancements aim to strengthen the model’s ability to extract and exploit fine-grained features, improve the model’s detection performance, and achieve high-precision wheat seedling detection.

### Construction of the wheat seedling datasets

**Fig. 2:**
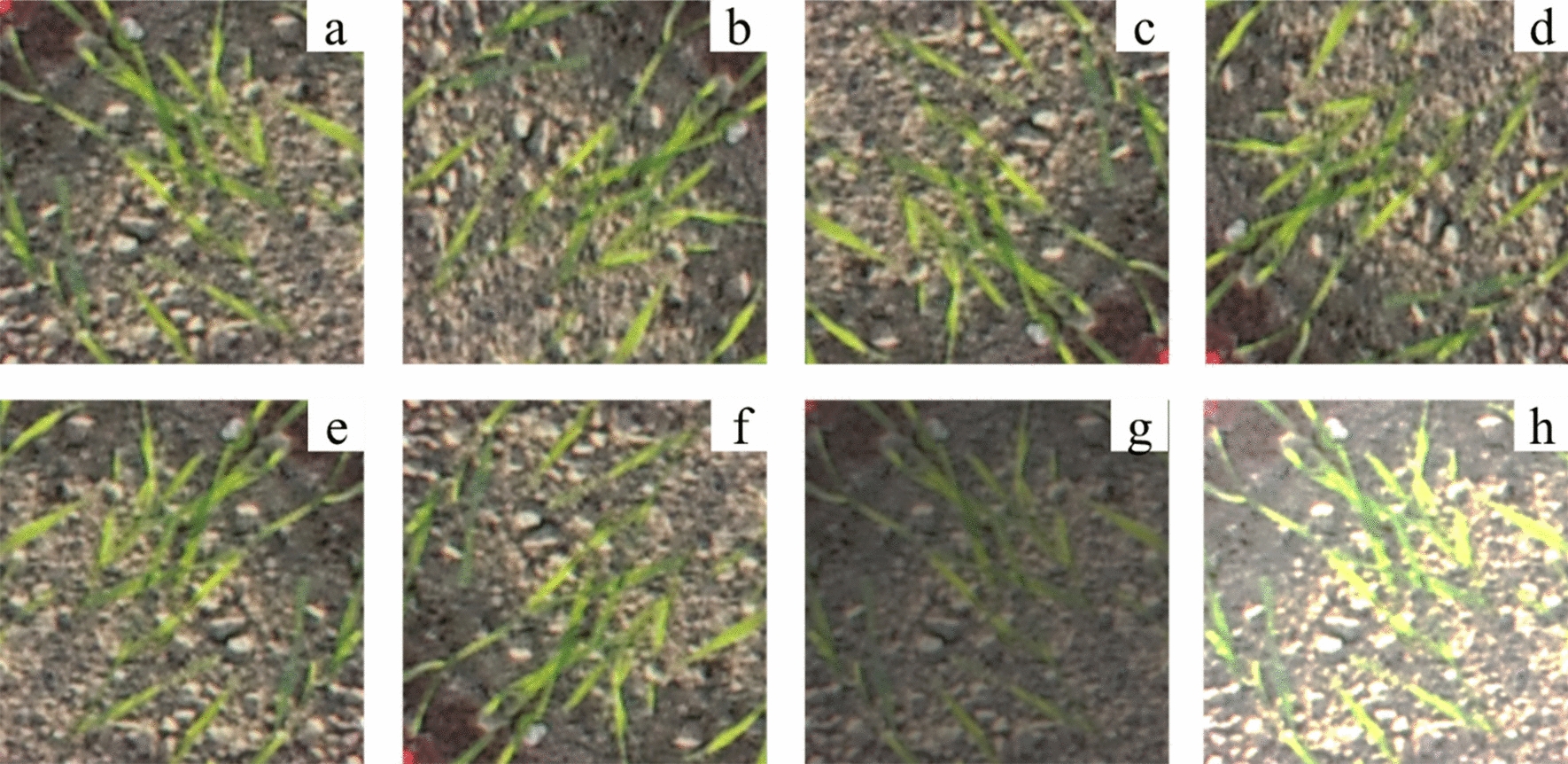
Data augmentation **a** the original image, **b** the original image rotated by 90°, **c** the original image rotated by 180°, **c** the original image rotated by 270°, **f** horizontal rotation **f** vertical rotation, **g** and **h** brightness balance

The experiment was conducted at Zhujiaqiao Village, Baipu Town, Rugao City, Jiangsu Province (120°46’ E, 32°16’ N) during the wheat seedling stage in 2021. A DJI™ MATRICE™ 210 drone with a DJI™ ZENMUSE™ X4S camera was used to capture RGB images of wheat seedlings at the seeding stage at 5 m high. Images were taken on the 30th day after sowing between 10:00 and 14:00. The drone flew at a constant speed of 2 m/s and stopped directly over the wheat seedlings to take pictures. The original image resolution was 5472 × 3648 pixels, and the images were segmented into 400 × 400-pixel patches to highlight the wheat seedling features and improve data processing efficiency. Data augmentation such as rotation (90°, 180°, 270°, and 360°), flipping, and brightness adjustment were applied to increase the diversity of the dataset and improve the robustness of the model during training (Fig. 2). The dataset for the study was increased from 1000 to 6000 images. These 6000 images were randomly shuffled and divided into training, validation, and testing sets in a ratio of 7:2:1. Furthermore, LabelImg [21] was used for image annotation.

### Image annotation methods

**Fig. 3:**
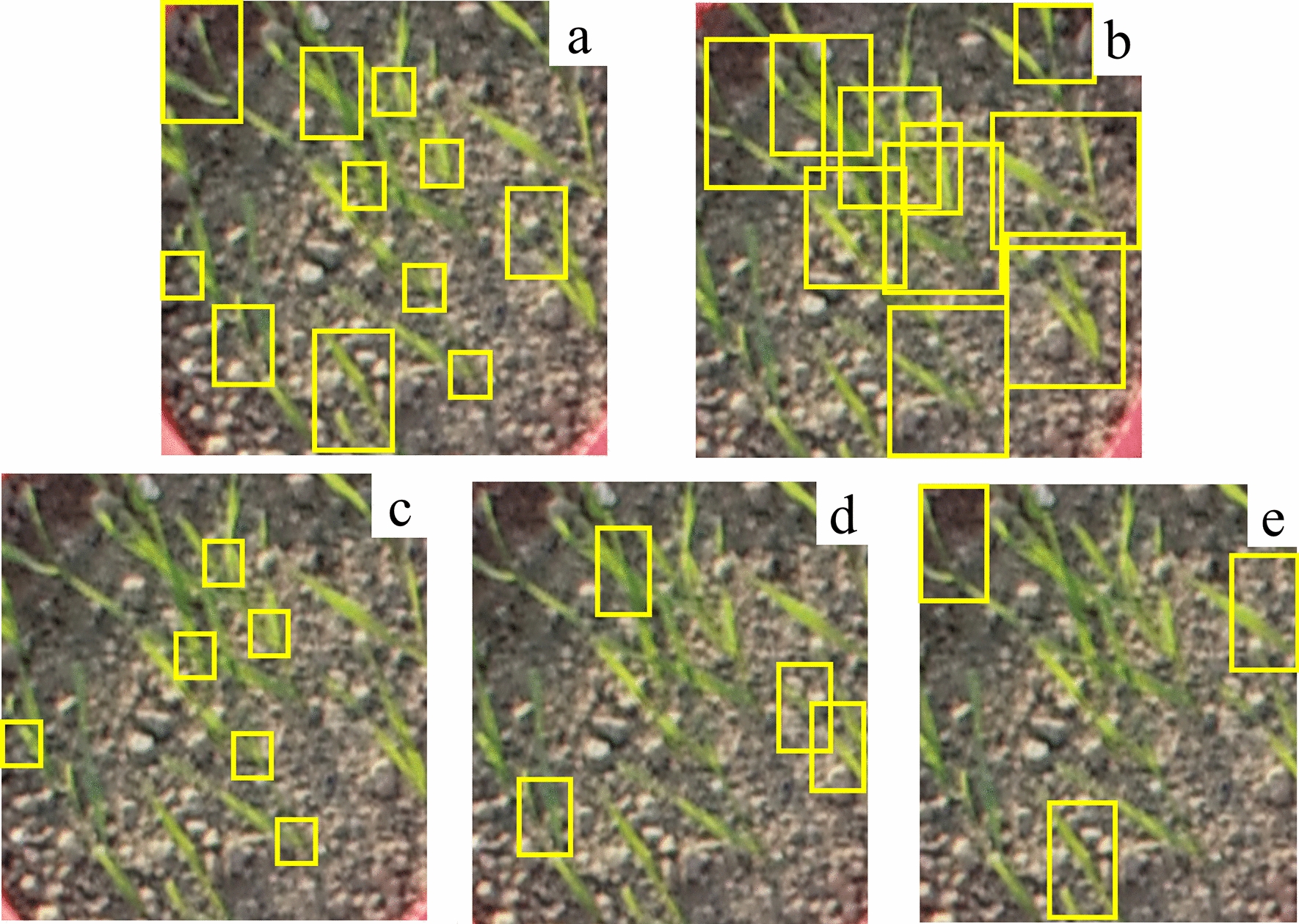
Wheat seedling images with different annotation modes: **a** local annotation, **b** global annotation, **c** small-size annotation, **d** medium-size annotation, **e** large-size annotation. Yellow boxes represent annotation

This study used two annotation modes, global annotation and local annotation (Fig. 3). The global annotation is based on the soil contact surface, and the whole two-leaf length of the wheat seedling is taken as an annotation box. The local annotation is drawn with the soil contact surface as the base and the stem of the wheat seedling as the origin, covering approximately one-third of the length between the two leaves of the wheat seedling.

To further explore the influence of different annotation frame sizes on the model detection accuracy, we designed and categorized annotation boxes of different sizes. In addition, due to the tolerance of the human visual system to degradations in image resolution, the annotation boxes were divided into three categories: small size (0–1000 pixels), medium size (1000–1500 pixels), and large size (greater than 1500 pixels) [22]. Furthermore, four different training strategies were constructed: using only the small-size annotated datasets, using the medium-size annotated datasets solely, using the large-size annotated datasets solely, and mixed datasets combining annotations of all sizes (Table 1, Fig. 3).

**Table 1:** Datasets of different annotation modes

Annotation	pixels	Number of images	Number of annotation boxes
Small	0–1000	5886	74970
Medium	1000–1500	3762	23058
Large	1500–3000	2226	9432
Muti-size	0–3000	6000	107460

### Improved wheat seedling detection model

#### Overview of YOLOv5

In this study, the YOLOv5 model was used as the baseline model [23]. YOLOv5 is a high-performance, one-stage, deep-learning object detection model that has been proven to be suitable for fusing modified modules [8, 14]. This study aims to explore the combination of wheat seedling features and annotation modes to construct a wheat seedling detection network. It can be applied to various single-stage object detection models, including YOLO series methods. In this regard, YOLOv5 was selected as a representative algorithm. YOLOv5 consists of three modules: the backbone module (Backbone), the neck module (Neck), and the detection module (Head).

**Fig. 4:**
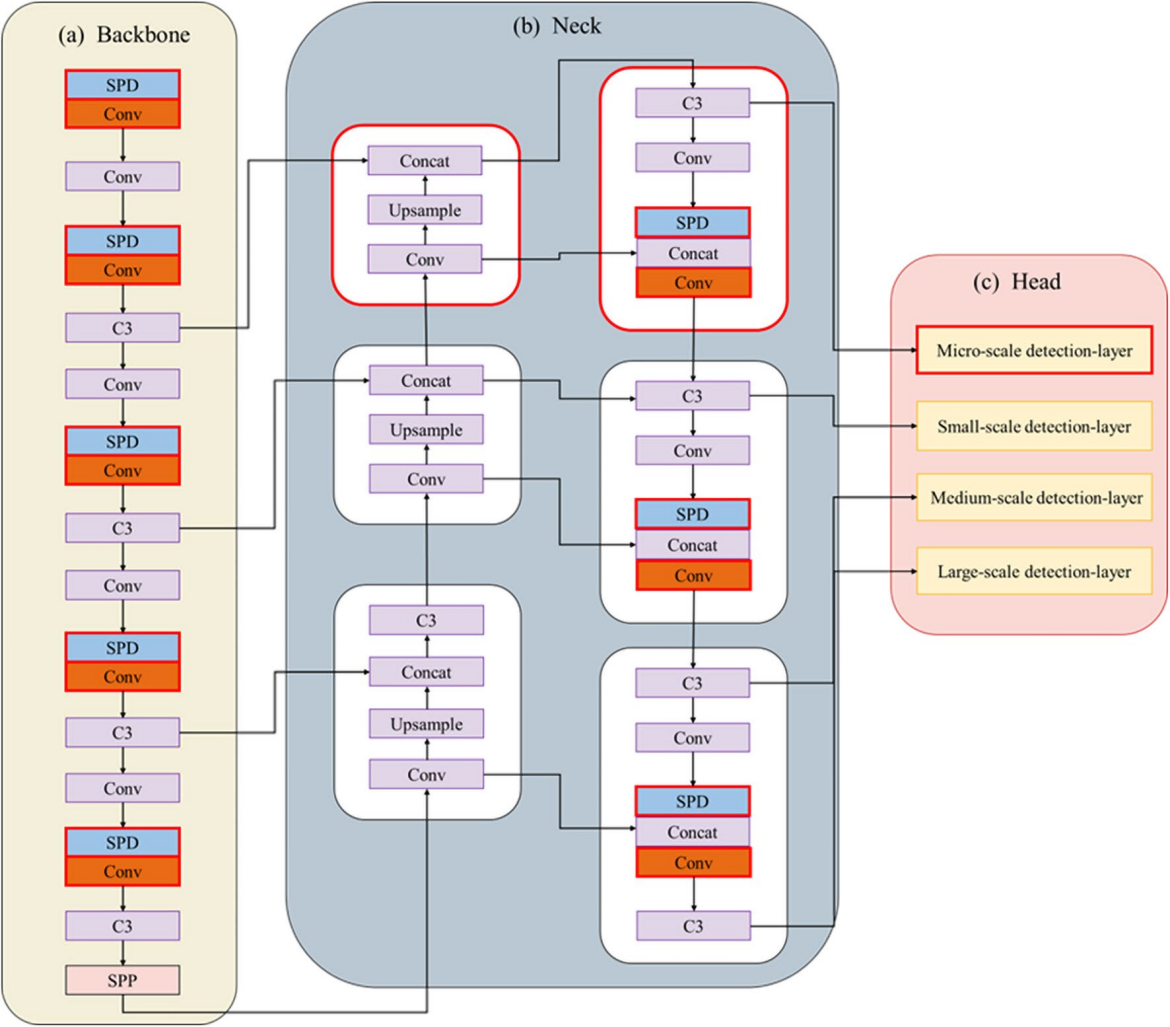
Improved YOLOv5 network architecture diagram. The red dashed box is the newly added micro-scale detection layer. The red solid line box is the SPD-Conv

In the standard YOLOv5, Both Backbone and Neck modules contain Convolutional (Conv) modules used to perform basic convolutional operations [24, 25]. The Head module consists of three detection layers responsible for object class and location prediction at three scales: small, medium, and large [26]. The proposed method adds a micro-scale detection layer to the head module, and the Space-to-depth Conv (SPD) module is integrated into both the backbone and neck modules. The introduction of SPD aims to fuse shallow spatial features with deep semantic features to obtain richer fine-grained feature information. The model is optimized by scaling the width and depth to retain all the discriminative feature information, resulting in an optimal wheat seedling detection model (Fig. 4).

#### Adding a micro-scale detection layer

The standard YOLOv5 includes large-scale, medium-scale, and small-scale detection layers that output feature maps with 1/32, 1/16, and 1/8 of the input image size, targeting large, medium, and small-sized objects [23]. However, due to the tiny size of the local annotation box of the wheat seedling in the images, the detection layers limit the capability of YOLOv5 to accurately detect the local region of the wheat seedling. This study proposed a strategy to incorporate a micro-scale detection layer by downsampling the input image dimensions by four [27]. This layer is designed to extract shallow spatial details and fuse them with deep semantic features, resulting in feature maps that are suitable for detecting tiny wheat seedling detection. These feature maps are 1/4 of the size of the input image. Integrating the micro-scale detection layer makes the network perform well in wheat seedling detection with local annotation.

#### Adding space-to-depth module

**Fig. 5:**
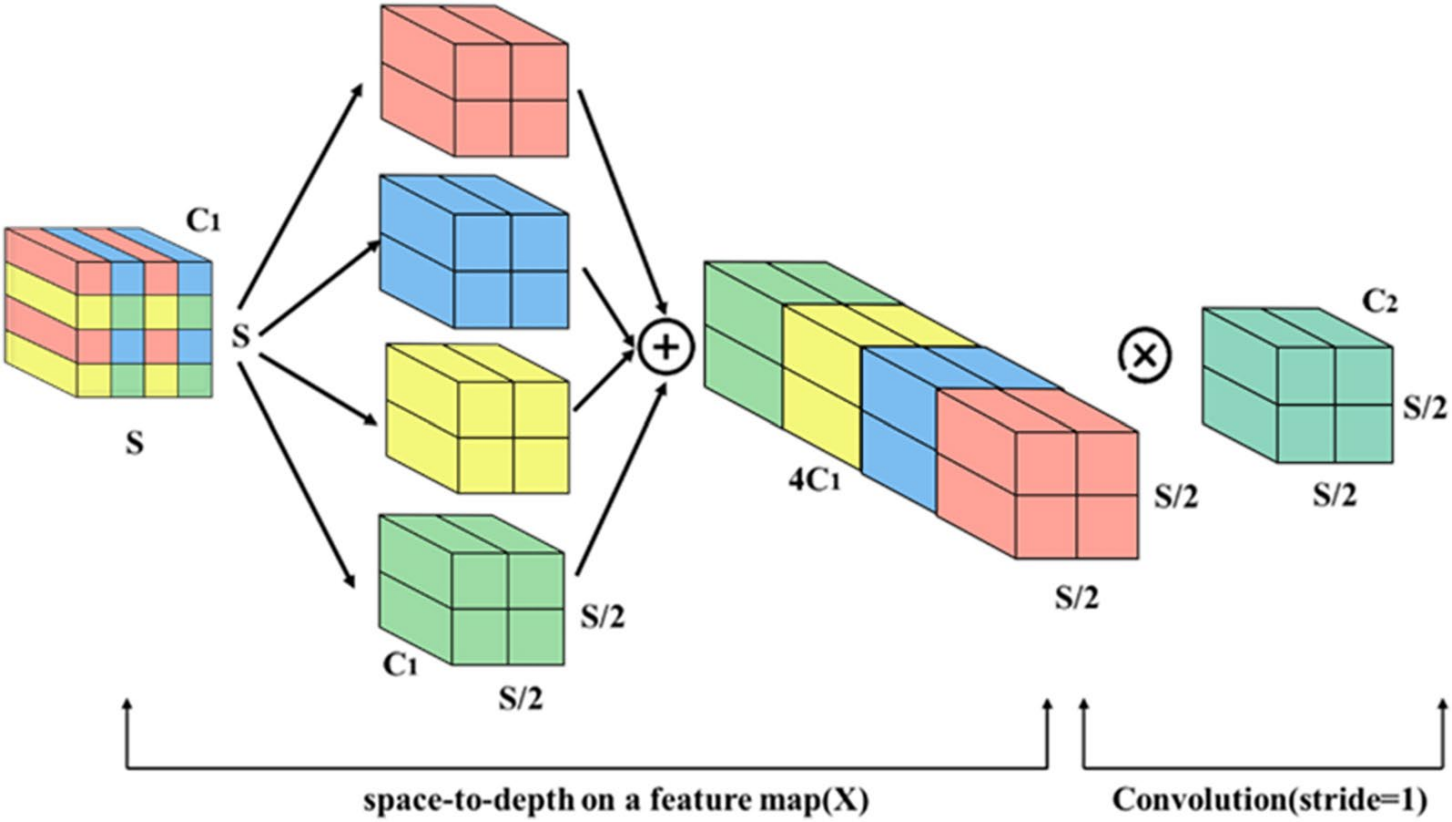
The feature processing with SPD-Conv

The SPD module was introduced into the standard YOLOv5 to enhance detection performance [28]. The SPD module uses dilated convolutions with different dilation rates to capture multiscale contextual information effectively [29]. Hence, the module can capture the wheat seedlings’ global and local features with dilated convolutions. The SPD module takes the feature map as input and performs downsampling within the entire neural network (Fig. 5) [28]. It generates four sub-feature maps by applying dilated convolutions with different dilation rates. These sub-feature maps are spatially concatenated to expand the preserved channel dimension and capture more detailed information. This process enriches the learning of fine-grained features for small-sized and densely occluded wheat seedlings, improving the accuracy and robustness of the detection model.

## Experiment and results

### Experiment configuration and training strategy

The experiments were conducted on a workstation with an Intel® Xeon® processor, 4 NVIDIA® Titan V graphics processing units (12 GB memory), and 500 GB memory. The operating system used was Ubuntu 16.06. For neural networks, the hyperparameters were manually adjusted based on model training results [30]. Since we focused on comparing different models in this research, we have kept the hyperparameters constant. The hyperparameters would be maintained at similar values to maintain consistency among all models [31]. Considering the collected dataset and the applied scene, YOLOv5n and YOLOv7-tiny were selected as the benchmark for YOLOv5 [23] and YOLOv7 [32], respectively. YOLOv3 [33], SSD [34], RetinaNet [35], and Faster-RCNN [36] were also selected to perform the experiment for comparison, and the hyperparameters are listed in Table 2. The batch size and training epochs were set by the number of images, image resolution, and computer hardware [37]. The learning rate, weight decay and momentum were set by the changes in loss during the model training process [38].

**Table 2:** Hyperparameters settings

Model	Batch size	Epoch	Learning rate	Weight decay	Momentum
YOLOv5	8	300	0.010	0.0005	0.9
YOLOv7	8	300	0.010	0.0005	0.9
YOLOv3	8	300	0.010	0.0005	0.9
SSD	8	300	0.008	0.0005	0.9
RetinaNet	8	300	0.012	0.0005	0.9
Faster-RCNN	8	300	0.009	0.0005	0.9

### Evaluation metrics

This study evaluated the model’s performance in detecting the local region of wheat seedlings from detection speed and detection accuracy. The detection speed refers to the number of detected images per second (FPS) [39, 40], and Precision(*P*), Recall(*R*), and Average Precision (*AP*) are used to evaluate the detection accuracy of the model:

1 $$P = \frac{TP}{{TP + FP}}$$

2 $$\begin{gathered} R = \frac{TP}{{TP + FN}} \hfill \\ \hfill \\ \end{gathered}$$

3 $$AP{ = }\int_{0}^{1} {} P(R)dR$$

According to the evaluation metrics for neural network models, the detection results can be classified into four classes: True Positive (*TP*), False Positive (*FP*), True Negative (*TN*), and False Negative (*FN*). If the Intersection over Union (IoU) between the detection box and the annotation box of the wheat seedling is greater than 0.5, it is considered a *TP*, indicating that the detection box correctly identifies the wheat seedling. If the IoU is less than 0.5, the box is marked as an *FP*, indicating that the detection box incorrectly identifies the background as a wheat seedling. If there is no corresponding detection box for a wheat seedling annotation box, it is labeled as an *FN*, indicating a missed detection of a wheat seedling. In this study, *TP* represents the number of correctly detected wheat seedlings, while *FP* represents the number of incorrectly detected wheat seedlings. *FN* represents the number of wheat seedlings that the model did not detect. *AP* is the average precision value within the range of recall rates from 0 to 1 for detecting a given class. *AP* comprehensively evaluates the model’s precision and recall to assess detection accuracy. A higher *AP* indicates a higher detection accuracy of the model [41, 42].

## Results

**Fig. 6:**
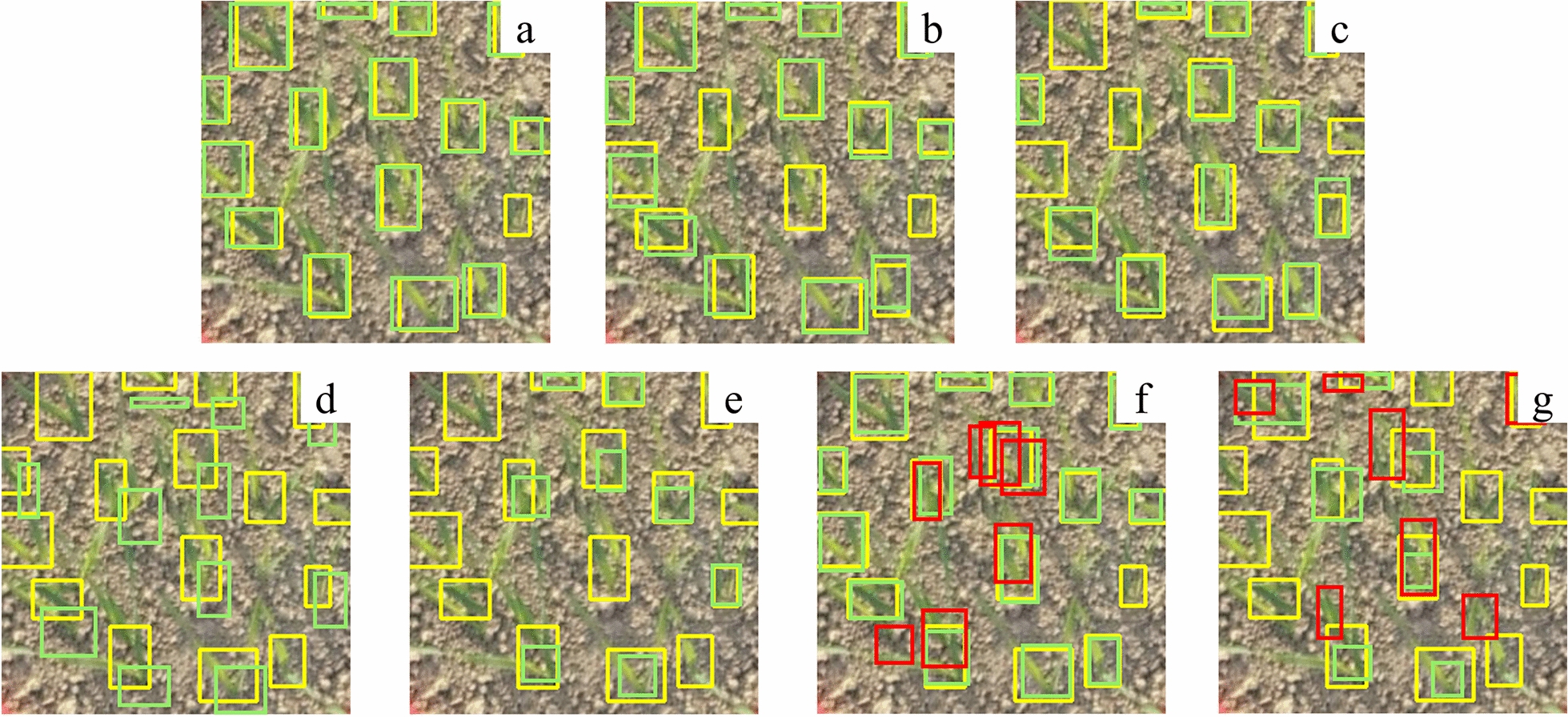
The proposed method and other state-of-the-art object detection results: **a** proposed, **b** YOLOv5, **c** YOLOv7, **d** YOLOv3, **e** SSD, **f** RetinaNet, **g** Faster-RCNN. Yellow boxes represent annotation, green boxes represent detection, and red boxes represent false detection

**Fig. 7:**
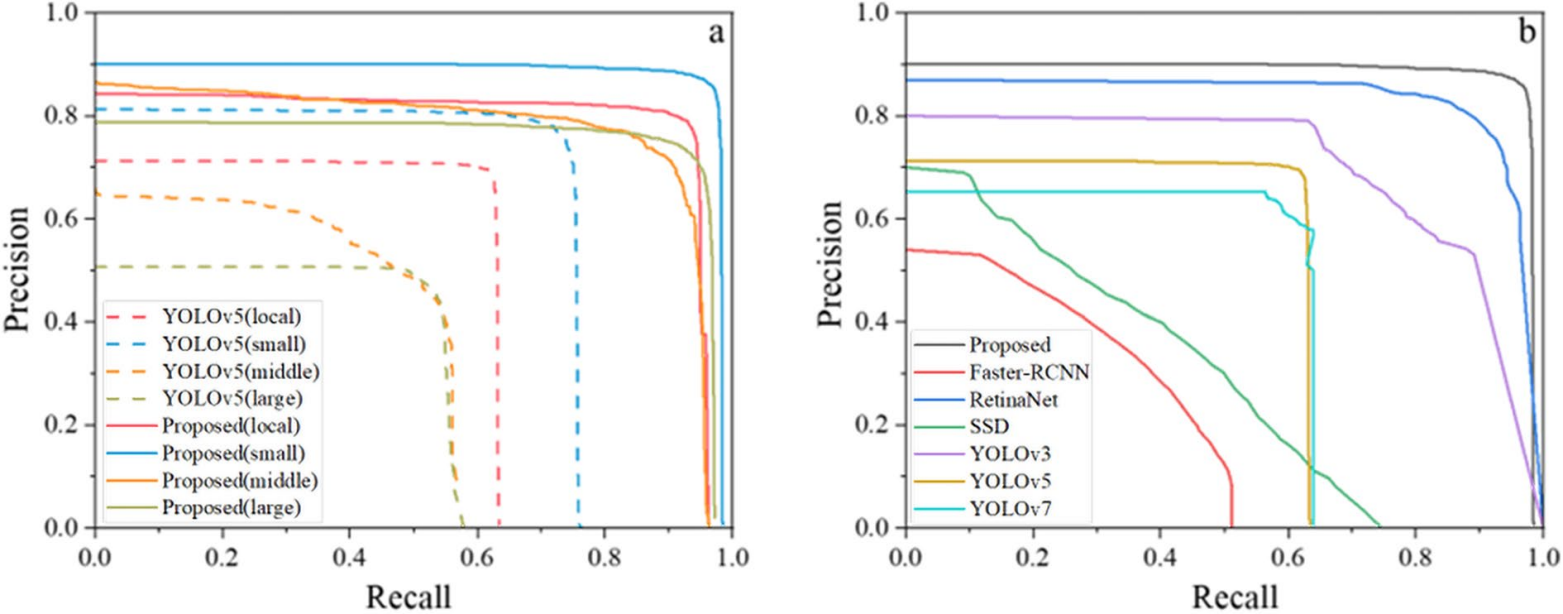
Precision and recall curves of wheat seedling detection: **a** the precision and recall curves of the proposed method and the standard YOLOv5 with different annotation modes, **b** the precision and recall curves of the proposed method and other object detection networks

The experimental results show that the proposed method achieves high accuracy in wheat seedling detection, and the annotation mode significantly influences the detection accuracy. The detection accuracy based on local annotation is 6.3% higher than that based on global annotation (Table 3, Fig. 6). Moreover, different sizes of local annotation boxes lead to different detection accuracies (Fig. 7), with the highest accuracy observed for small-sized annotation boxes. Fusing the proposed optimized detection model and small-sized local annotation mode is the most effective improvement, increasing *AP* by 3.7% and 13.5% compared to medium-sized and large-sized annotation boxes, respectively (Table 3). The optimized model outperforms YOLOv5, YOLOv7, and other object detection methods, significantly improving the accuracy of wheat seedling detection without significantly reducing the detection speed (Table 4, Fig. 6). The standard YOLOv5 achieves detection accuracy of 74.5%, 63.7%, and 30.2% for small-sized, medium-sized, and large-sized annotation datasets. In contrast, the optimized model achieves accuracies of 90.1%, 86.3%, and 76.5% for the respective datasets, representing improvements of 15.6, 22.6, and 46.3 percentage points compared to the standard YOLOv5.

**Table 3:** Comparison of the detection accuracy between the original YOLOv5 and the proposed method on the wheat seedling dataset with different annotations

Annotation	Training Datasets	Testing Datasets	Method	AP
Local annotation	Small size	All-size	YOLOv5 Proposed	74.5% 90.1%
	Medium size	All-size	YOLOv5 Proposed	63.7% 86.3%
	Large size	All-size	YOLOv5 Proposed	30.2% 76.5%
	Muti-size	All-size	YOLOv5 Proposed	66.5% 84.6%
Global annotation	All-size	All-size	YOLOv5	60.2%

**Table 4:** Comparison between the proposed method and other state-of-the-art object detection networks

Method	Image size	AP	FPS	Params (M)
Proposed	400 × 400	90.1%	35	2.6
YOLOv5	400 × 400	66.5%	30	1.9
YOLOv7	400 × 400	61.3%	28	6.2
YOLOv3	400 × 400	63.0%	25	61.5
SSD	400 × 400	68.3%	19	24.4
RetinaNet	400 × 400	65.4%	15	93.8
Faster-RCNN	400 × 400	60.2%	17	98.8

### Ablation experiment

Ablation experiments evaluated the proposed modules’ effectiveness, feasibility, and optimization effects, including the micro-scale detection layer and the Space-to-depth Conv. We also considered the effect of the dataset and the model’s hyperparameter settings, and the operating environment’s consistency was maintained. The results indicate that the proposed modules have a positive impact (Table 5). Among these, the datasets of local annotation mode have the most significant influence, leading to an 8% increase in *AP*. Combining the improvements in the annotation mode and model structure, the *AP* reaches 90.1%. This finding highlights the importance of optimizing both the dataset annotation and the model architecture to achieve better performance in wheat seedling detection.

**Table 5:** Ablation experiment results

Small size datasets	Micro-scale detection layer	Space-to-depth Conv	AP (%)
			66.5
√			74.5
	√		70.1
		√	72.3
√	√		85.5
√		√	86.6
	√	√	73.7
√	√	√	90.1

## Discussion

**Fig. 8:**
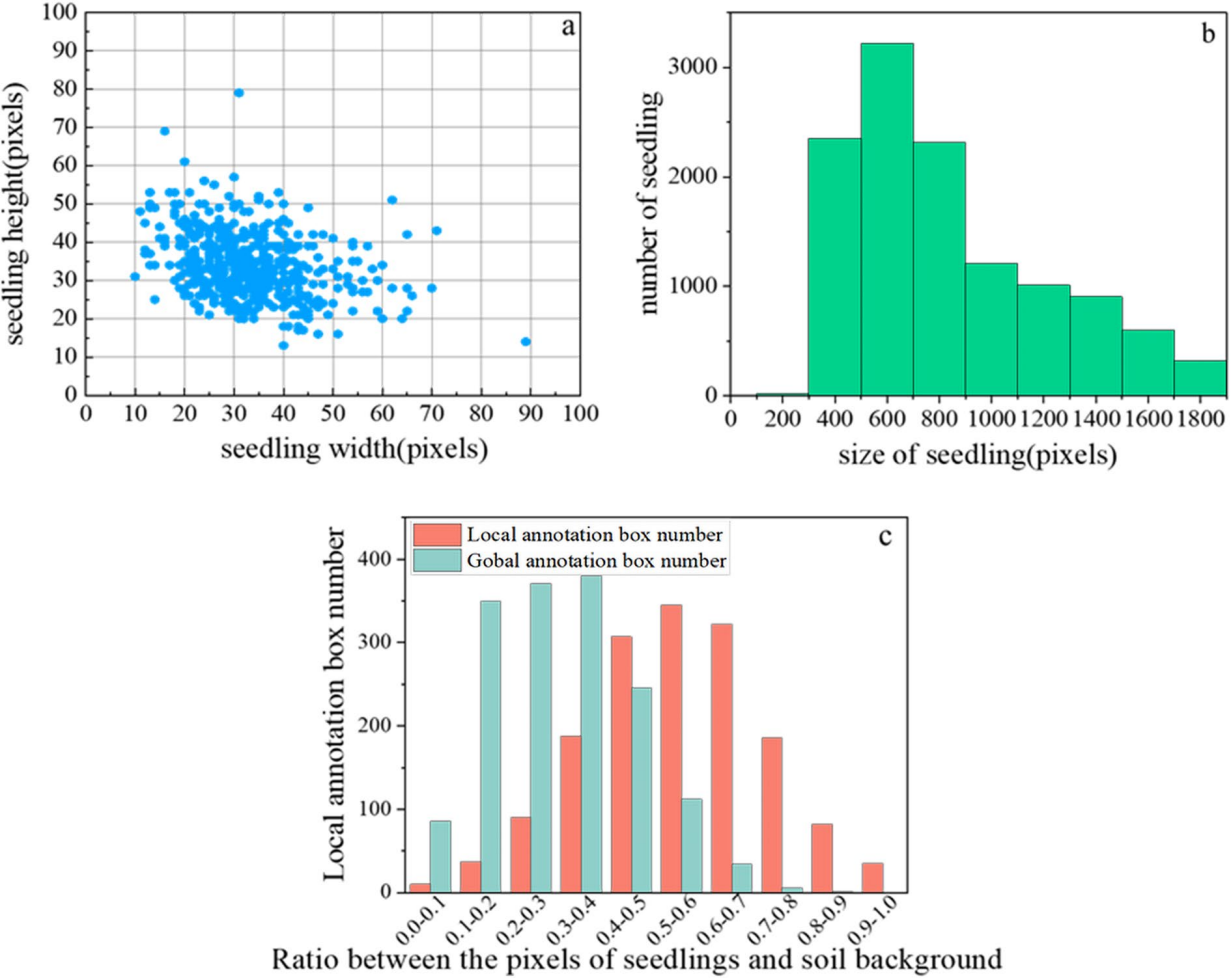
The distribution of local annotation of wheat seedlings in UAV images: **a** size distribution of wheat seedling length and width, **b** the number distribution of different sizes of wheat seedlings, **c** the ratio between the pixels of seedlings and soil background in local annotation mode and global annotation mode

The study suggests replacing the global annotation of wheat seedlings with local annotation mode, which further enhances the detection performance of the model. Wheat seedlings vary in size in the field. Accurate detection of wheat seedlings is crucial for convolutional neural networks. The local annotation mode can highlight wheat seedlings’ size characteristics and reduce manual annotation difficulty [43–45]. One-stage methods often have poor detection performance due to background class imbalance in densely distributed fields [19]. Wheat seedlings have complex and diverse shapes with severe overlapping. Huge annotation boxes can weaken the network’s performance [17, 46]. The proposed local annotation mode defines the boundary range of wheat seedlings accurately. This mode balances the proportion between wheat seedlings and the soil background in the image and removes a significant amount of irrelevant information within the annotation boxes. It reduces the overlap between annotation boxes and improves the detection performance effectively. Replacing the global annotation mode with the local annotation mode for wheat seedlings significantly reduces the annotation area. Approximately 80% of the total number of annotation boxes range from 400 to 1000 pixels in size (Fig. 8). Under different annotation modes, the ratio of wheat to soil background pixels is lower for the global annotation mode compared to the local annotation mode for wheat seedlings (Fig. 8). The number of wheat seedling pixels in the image is much smaller than the background, resulting in the detector training process without a desired accuracy [47]. In the local annotation mode of wheat seedling datasets, the detection accuracy of the small-sized annotation datasets is higher than that of the medium-sized and large-sized annotation datasets (Table 3). These results show that smaller annotation boxes can increase the ratio of the pixels of wheat seedlings to the soil background. Therefore, the proposed local annotation mode can improve detection accuracy.

**Fig. 9:**
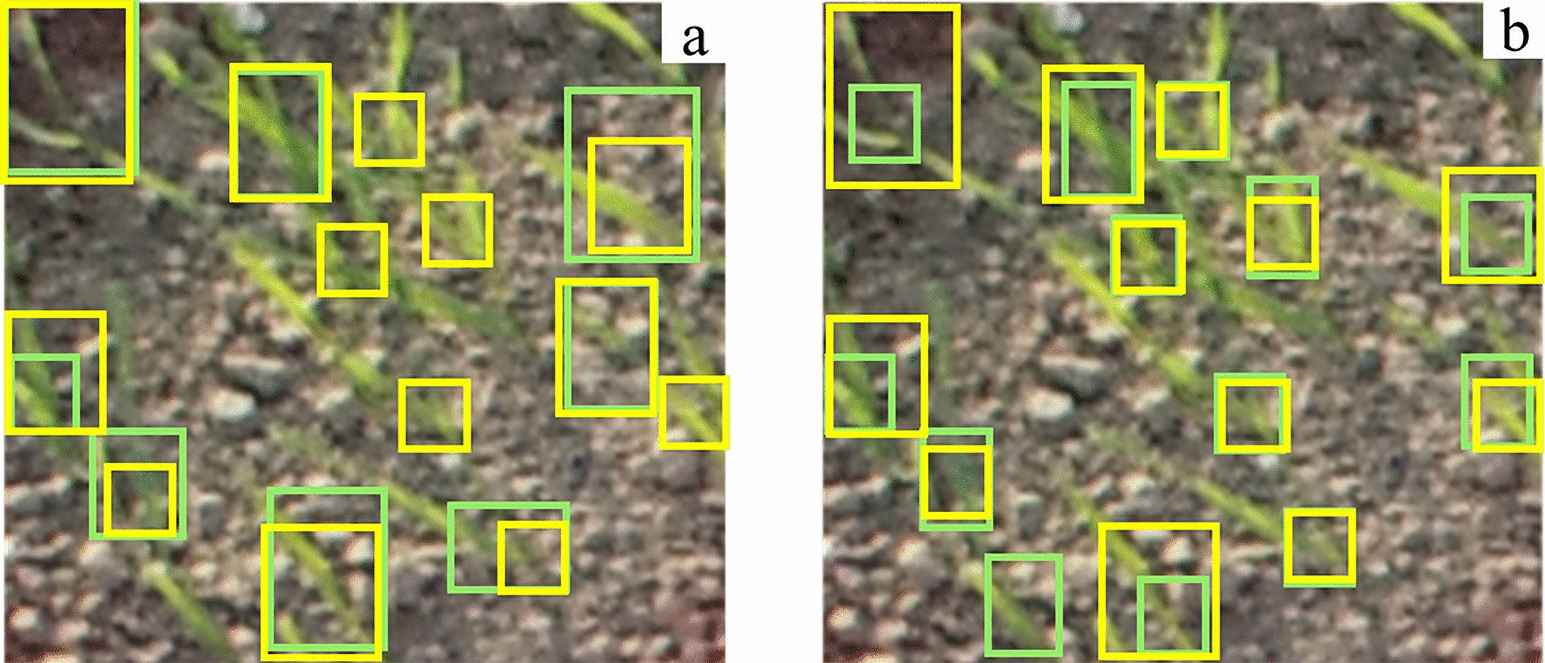
Detection results of the model without the SPD-Conv module (**a**) and with the SPD-Conv module (**b**). Yellow boxes represent annotation, and green boxes represent detection

Adopting the local annotation for wheat seedlings detection proposes further requirements for the model construction [48–50] and requires a careful balance and improvement of the model architecture. Indeed, the architecture of the model and annotation mode both influence the accuracy of the model [45, 51, 52]. The combination of an appropriate model architecture and a suitable annotation mode plays a crucial role in the model’s overall performance. Existing wheat seedling detection methods based on deep learning rely on CNN modules for effective feature extraction. However, the receptive field of the CNN is limited by the size of the convolutional kernel and the depth of the network, which can result in a lack of specificity and generalizability [53, 54]. Hence, the proposed method extracts more detailed information and effectively integrates multiscale features to improve the detection performance of small-sized wheat seedlings with the local annotation mode by applying the SPD-Conv module to YOLOv5, significantly improving the model’s detection performance. The SPD-Conv module performs spatial dimension concatenation, expanding the preserved channel dimension to retain more detailed information. It improves the Neck and Backbone in feature extraction, effectively fusing feature information from multiple scales, and leads to better detection accuracy on wheat seedlings (Fig. 9).

**Fig. 10:**
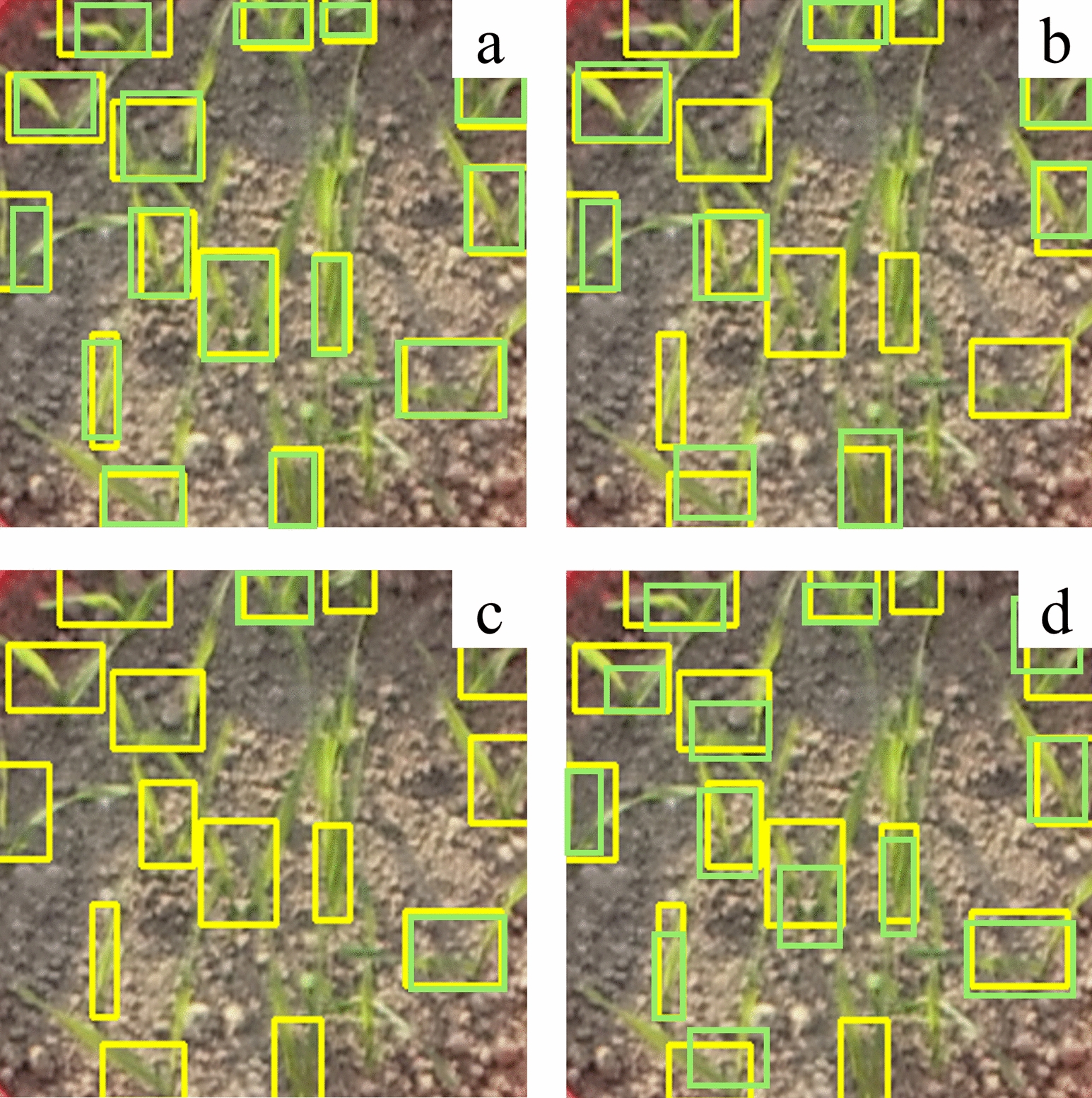
**a** small-size annotation boxes and detection boxes, **b** medium-size annotation boxes and detection boxes, **c** large-size annotation boxes and detection boxes, **d** multi-size annotation boxes and detection boxes. Yellow boxes represent annotation, and green boxes represent detection

**Fig. 11:**
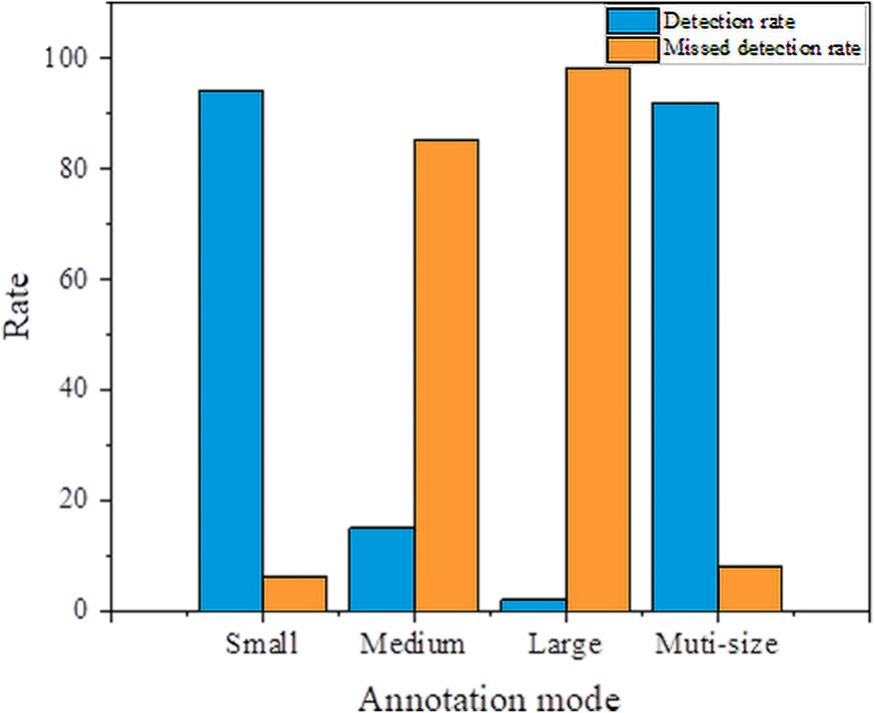
The detection rate and missed detection rate during the neural network training process based on the small, medium, and large size annotated boxes of local annotation mode of wheat seedling

In addition, this study improves feature extraction for shallow spatial details by incorporating a micro-scale detection layer [27]. The extracted features are then fused with deep semantic features to produce feature maps tailored for detecting small-sized wheat seedlings (Fig. 9). There is a detection imbalance for positive samples in the training process. The detection layer of the model shows varying quantities and qualities of positive samples in the output results for small, medium, and large-sized objects (Fig. 10). The detection rate of small annotation mode, medium annotation mode, large annotation mode, and muti-size annotation mode reached 94%, 15%, 2% and 92% respectively (Fig. 11). The number of positive samples for small-sized objects is higher than for medium-sized and large-sized objects, resulting in the highest detection rate and the lowest missed rate for small-sized wheat seedlings. The number of seedlings is a crucial indicator of the plant population during the seedling stage. False seedling detection would affect grain structure and cause wrong predictions of wheat yield [18, 20]. The experimental results show that the model proposed has a more robust feature extraction capability for small objects compared to the standard YOLOv5. The improved model outperforms other models and significantly improves the global annotation accuracy for the images (Table 3).

In the field, wheat seedlings can be affected by weeds and other plants [55, 56]. These disturbances can be very similar to wheat seedlings, challenging the detection process and leading to error detection. This study investigated the impact of different-sized annotation boxes under the local annotation mode of wheat seedlings and refined the model structure to address the mentioned errors (Table 5). In future work, we plan to consider complex field conditions such as environmental factors, lighting variations, and weed interference. We will expand the dataset to include a broader range of wheat seedlings in the farmland environment and develop a more robust wheat seedling detection method under various field conditions.

## Conclusion

This study investigates the impact of annotation modes on the detection performance of the deep learning model for wheat seedlings and determines an optimized local annotation strategy. Moreover, we refined the YOLOv5 structure to match the local annotation mode by adding a micro-scale detection layer and integrating the SPD-Conv module. The results show that the fusion of local annotation mode and refined model structure can significantly improve wheat seedling detection accuracy. The proposed method extends the applicability of the YOLO to wheat seedling detection under occlusion and overlapping field conditions. It provides a highly informative and practical method for wheat seedling detection and solid references for future research and applications in this area.

## Data Availability

The datasets generated and/or analyzed during the current study are available from the corresponding author upon reasonable request.

## References

[1] Zhang P, Li D (2023). Automatic counting of lettuce using an improved YOLOv5s with multiple lightweight strategies. Expert Syst Appl.

[2] Maharana K, Mondal S, Nemade B (2022). A review: data pre-processing and data augmentation techniques. Global Transit Proc.

[3] Pan Y, Zhu N, Ding L (2022). Identification and counting of sugarcane seedlings in the field using improved faster R-CNN. Remote Sensing.

[4] Kumar D, Kukreja V (2022). Deep learning in wheat diseases classification: a systematic review. Multimedia Tools Appl.

[5] Ashqar BA, Abu-Nasser BS, Abu-Naser SS. Plant seedlings classification using deep learning. International Journal of Academic Information Systems Research (IJAISR). 2019; 3(1): 7-14.

[6] Ofori M, El-Gayar OF. Towards deep learning for weed detection: deep convolutional neural network architectures for plant seedling classification. In Proceedings of the Americas Conference on Information Systems. Salt Lake City, UT, USA, 10–14 August 2020.

[7] Yang S, Luo P, Loy C C, et al. Wider face: a face detection benchmark. Proceedings of the IEEE conference on computer vision and pattern recognition. 2016: 5525-5533. https://doi.org/10.48550/arXiv.1511.06523

[8] Wang Y, Qin Y, Cui J (2021). Occlusion robust wheat ear counting algorithm based on deep learning[J]. Front Plant Sci.

[9] Madec S, Jin X, Lu H (2019). Ear density estimation from high resolution RGB imagery using deep learning technique. Agric Forest Meteorol.

[10] Everingham M, Van Gool L, Williams CK (2010). The pascal visual object classes (voc) challenge. Int J Comput Vision.

[11] Russakovsky O, Deng J, Huang Z, et al. Detecting avocados to zucchinis: what have we done, and where are we going?. Proceedings of the IEEE international conference on computer vision. 2013.

[12] Deng J, Dong W, Socher R, et al. Imagenet: A large-scale hierarchical image database. 2009 IEEE conference on computer vision and pattern recognition. Ieee. 2009. 10.1109/CVPR.2009.5206848

[13] Liu H, Jiao L, Wang R (2022). WSRD-Net: a convolutional neural network-based arbitrary-oriented wheat stripe rust detection method. Front Plant Sci.

[14] Zhao J, Yan J, Xue T (2022). A deep learning method for oriented and small wheat spike detection (OSWSDet) in UAV images. Comput Electr Agric.

[15] Li J, Wang E, Qiao J (2023). Automatic rape flower cluster counting method based on low-cost labelling and UAV-RGB images[J]. Plant Methods.

[16] Ma H, Zhao W, Ji J, et al. A quick counting method for winter wheat at the seedling stage in fields based on an improved YOLOV4 model. Journal of Animal & Plant Sciences, 32(6): 2022, 1666-1681. https://doi.org/10.36899/JAPS.2022.6.0575

[17] Dong J, Lee J, Fuentes A (2022). Data-centric annotation analysis for plant disease detection: strategy, consistency, and performance. Front Plant Sci.

[18] Liu T, Wu W, Chen W (2016). Automated image-processing for counting seedlings in a wheat field. Precision Agric.

[19] Lin T-Y, Goyal P, Girshick R, et al. Focal loss for dense object detection. Proceedings of the IEEE international conference on computer vision. 2017. 10.1109/TPAMI.2018.2858826 30040631

[20] Guo X, Ge Y, Liu F (2023). Identification of maize and wheat seedlings and weeds based on deep learning. Front Earth Sci.

[21] Cgvict. roLabelImg. https://github.com/cgvict/roLabelImg. Accessed 1 May 2023.

[22] Torralba A, Fergus R, Freeman WT (2008). 80 million tiny images: a large data set for nonparametric object and scene recognition. IEEE Trans Pattern Anal Machine Intell.

[23] Ultralytics. YOLOv5. https://github.com/ultralytics/yolov5. Accessed 1 May 2023.

[24] Chen Y, Zhang C, Qiao T, et al. Ship detection in optical sensing images based on YOLOv5. Twelfth International Conference on Graphics and Image Processing (ICGIP 2020), 2021. SPIE. 10.1117/12.2589395.

[25] Fang J, Liu Q, Li J. A deployment scheme of YOLOv5 with inference optimizations based on the triton inference server. 2021 IEEE 6th International Conference on cloud computing and big data analytics (ICCCBDA), 2021. IEEE. 10.1109/ICCCBDA51879.2021.9442557.

[26] Zhu X, Lyu S, Wang X, et al. TPH-YOLOv5: Improved YOLOv5 based on transformer prediction head for object detection on drone-captured scenarios. Proceedings of the IEEE/CVF international conference on computer vision. 2021.

[27] Zhao J, Zhang X, Yan J (2021). A wheat spike detection method in UAV images based on improved YOLOv5. Remote Sensing.

[28] Sunkara R, Luo T. 2022. No more strided convolutions or pooling: a new CNN building block for low-resolution images and small objects. Joint European Conference on Machine Learning and Knowledge Discovery in Databases. Springer. 10.1007/978-3-031-26409-2_27.

[29] Zhao W, Liu S, Li X (2022). Fast and accurate wheat grain quality detection based on improved YOLOv5. Comput Electron Agric.

[30] Su Y, Liu Q, Xie W (2022). YOLO-LOGO: a transformer-based YOLO segmentation model for breast mass detection and segmentation in digital mammograms. Comput Methods Programs Biomed.

[31] Azevedo P, Santos V (2024). Comparative analysis of multiple YOLO-based target detectors and trackers for ADAS in edge devices. Robotics Autonomous Syst.

[32] Wang C-Y, Bochkovskiy A, Liao H-Y M. YOLOv7: Trainable bag-of-freebies sets new state-of-the-art for real-time object detectors. Proceedings of the IEEE/CVF Conference on Computer Vision and Pattern Recognition. 2023.

[33] Redmon J, Farhadi A (2018). Yolov3: an incremental improvement. arXiv.

[34] Liu W, Anguelov D, Erhan D (2016). SSD: single shot multibox detector computer vision–ECCV, 14th European Conference.

[35] Wang Y, Wang C, Zhang H (2019). Automatic ship detection based on RetinaNet using multi-resolution Gaofen-3 imagery. Remote Sensing.

[36] Ren S, He K, Girshick R, et al. Faster r-cnn: towards real-time object detection with region proposal networks. Advances in neural information processing systems, 2015;28. 10.1109/TPAMI.2016.2577031 27295650

[37] Justus D, Brennan J, Bonner S (2018). Predicting the computational cost of deep learning models. IEEE international conference on big data (Big Data). IEEE.

[38] Smith LN (2018). A disciplined approach to neural network hyper-parameters: Part 1–learning rate, batch size, momentum, and weight decay. arXiv.

[39] Tatbul N, Lee T J, Zdonik S, et al. Precision and recall for time series. arXiv preprint. 2018.10.48550/arXiv.1803.03639.

[40] Ishii I, Ichida T, Gu Q (2013). 500-fps face tracking system. J Real-Time Image Proc.

[41] Yang S, Luo P, Loy C-C, et al. From facial parts responses to face detection: A deep learning approach. Proceedings of the IEEE international conference on computer vision, 2015.

[42] Zheng YY, Kong JL, Jin XB (2019). CropDeep: the crop vision dataset for deep-learning-based classification and detection in precision agriculture. Sensors.

[43] Zhang Y, Ling H, Gao J, et al. Datasetgan: efficient labeled data factory with minimal human effort. Proceedings of the IEEE/CVF Conference on Computer Vision and Pattern Recognition. 2021.

[44] Qu H, Wu P, Huang Q (2020). Weakly supervised deep nuclei segmentation using partial points annotation in histopathology images. IEEE Trans Med Imaging.

[45] Ke X, Zhou M, Niu Y (2017). Data equilibrium based automatic image annotation by fusing deep model and semantic propagation. Pattern Recogn.

[46] Gardner M, Artzi Y, Basmova V (2020). Evaluating ’models’ local decision boundaries via contrast sets. arXiv.

[47] Schmarje L, Grossmann V, Zelenka C (2022). Is one annotation enough?—A data-centric image classification benchmark for noisy and ambiguous label estimation. Adv Neural Inf Process Syst.

[48] Bird S, Day D, Garofolo J (2000). ATLAS: a flexible and extensible architecture for linguistic annotation. Proc Second Int Conf Lang Resour Eval.

[49] Harrison NB, Avgeriou P (2010). How do architecture patterns and tactics interact? A model and annotation. J Syst Software.

[50] Fill HG, Schremser D, Karagiannis D (2013). A generic approach for the semantic annotation of conceptual models using a service-oriented architecture. Int J Knowledge Manag.

[51] Lin KL, Lo CK, Tsay RS (2010). Source-level timing annotation for fast and accurate TLM computation model generation. 15th Asia and South Pacific Design Automation Conference (ASP-DAC). IEEE.

[52] Smith AG, Han E, Petersen J (2022). RootPainter: deep learning segmentation of biological images with corrective annotation. New Phytol.

[53] Yadhav SY, Senthilkumar T, Jayanthy S (2020). Plant disease detection and classification using CNN model with optimized activation function. International conference on electronics and sustainable communication systems (ICESC). IEEE.

[54] Arkin E, Yadikar N, Xu X (2023). A survey: object detection methods from CNN to transformer. Multimedia Tools Appl.

[55] Soltani A, Gholipoor M, Zeinali E (2006). Seed reserve utilization and seedling growth of wheat as affected by drought and salinity. Environ Exp Bot.

[56] Mbũgwa GW, Krall JM, Legg DE (2012). Interference of Tifton burclover residues with growth of burclover and wheat seedlings. Agronomy J.

